# Chronicle of a Health Crisis: Global Implications of the 2008 Melamine Event

**DOI:** 10.1289/ehp.117-a553b

**Published:** 2009-12

**Authors:** Tanya Tillett

**Affiliations:** **Tanya Tillett**, MA, of Durham, North Carolina, is a staff writer/editor for *EHP.* She has been on the *EHP* staff since 2000 and has represented the journal at national and international conferences

Melamine is commonly used to manufacture strong and durable laminates, plastics, adhesives, and flame-resistant textiles. It also has been deliberately added to food and animal feed, sometimes in high amounts, to boost the appearance of protein content based on nitrogen analysis. The result can be serious health threats, including renal failure and death. In 2007–2008, for instance, the deliberate addition of melamine to raw milk used in powdered infant formula and other milk and dairy products caused an outbreak of kidney stones and renal failure in Chinese infants and raised significant implications for global food and feed safety **[*EHP*** **117:1803–1809; Gossner et al.]**.

Because melamine is used in such a wide range of products, its trace presence in many foods is inevitable—action is not usually taken if levels are below 1.0 mg/kg for infant foods or 2.5 mg/kg for other food products. In comparison, contaminated powdered infant formula produced by the Sanlu Group and distributed in China contained up to 2,563 mg/kg.

Using information originally reported by the Chinese Ministry of Health to the World Health Organization and shared through the International Food Safety Authorities Network (INFOSAN), the authors describe the unfolding of events from the first reported cases of sick babies in China to the export of contaminated dairy and non-dairy products (including ammonium bicarbonate, fresh and dried eggs, nondairy creamer, and animal feed) that eventually reached 47 known countries. Although parents who used Sanlu formula first began filing complaints in December 2007, the global community did not become aware of the crisis until September 2008.

Countries responded in a variety of ways ranging from taking no action at all to banning all imports of milk and dairy products from China. Meanwhile, China reported a total of 6 child deaths and 294,000 cases of children affected by consumption of contaminated formula and milk products. Health effects ranged from discolored urine to kidney stones to acute renal failure and subsequent death. Because milder cases were often asymptomatic, many more children may have been affected both in China and abroad.

Given the potential global impact of the 2008 event, the authors state that well-structured national food safety systems—combined with coordination among food safety authorities and rapid communication through INFOSAN—are key components in controlling such outbreaks. There also should be one harmonized set of international standards for acceptable levels of potential contaminants in food and feed products as well as universal methods of detection, prevention, and containment.

## Figures and Tables

**Figure:**
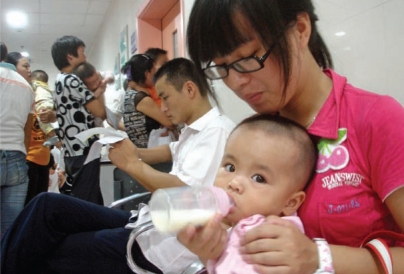
Chinese parents wait to have their babies examined for melamine effects at a Suzhou hospital, 22 September 2008.

